# Clinical Evaluation of the Effectiveness of Digital Peroral Cholangioscopy‐Guided Lithotripsy for Difficult Bile Duct Stones

**DOI:** 10.1002/jhbp.70138

**Published:** 2026-05-29

**Authors:** Hirohito Minami, Shuntaro Mukai, Takayoshi Tsuchiya, Reina Tanaka, Ryosuke Tonozuka, Kazumasa Nagai, Hiroyuki Kojima, Noriyuki Hirakawa, Kyoko Asano, Takao Itoi

**Affiliations:** ^1^ Department of Gastroenterology and Hepatology Tokyo Medical University Tokyo Japan

**Keywords:** difficult bile duct stones, electrohydraulic lithotripsy, peroral cholangioscopy, YAG‐laser

## Abstract

**Background and Aims:**

Peroral cholangioscopy‐guided lithotripsy (POCS‐L) has emerged as a treatment option for difficult bile duct stones (DBDSs), but detailed evidence remains limited. This study evaluated its effectiveness, safety, and risk factors for adverse events (AEs).

**Methods:**

We retrospectively analyzed 132 consecutive patients who underwent POCS‐L for DBDSs between December 2014 and July 2023 at Tokyo Medical University Hospital. Electrohydraulic lithotripsy or holmium: yttrium‐aluminum‐garnet laser lithotripsy was performed under cholangioscopic guidance. The primary outcomes were technical and clinical success, and secondary outcomes included procedure‐associated AEs and risk factors for cholangitis.

**Results:**

The technical and clinical success rates were 98.5% and 94.7%, respectively, with a median procedure time of 56 min. Clinical success was higher for common bile duct stones than intrahepatic bile duct stones (98.0% vs. 83.9%). Procedure‐associated AEs occurred in 29.5% of patients, most commonly transient fever (16.7%) and cholangitis (11.4%). Multivariable analysis identified a stone diameter > 20 mm (odds ratio [OR]: 4.79; *p* = 0.016) and absence of postprocedural biliary drainage (OR: 3.73; *p* = 0.048) as independent predictors of cholangitis.

**Conclusions:**

POCS‐L is an effective and safe treatment for DBDSs. Postprocedural biliary drainage may reduce the risk of cholangitis, particularly in patients with large stones.

## 1 Introduction

Endoscopic retrograde cholangiopancreatography (ERCP) has been used as the primary treatment technique for bile duct stones. Most bile duct stones can be removed by the standard ERCP technique using a basket catheter, balloon catheter, or mechanical lithotripter following endoscopic sphincterotomy (EST), endoscopic papillary balloon dilation (EPBD), and endoscopic papillary large‐balloon dilation (EPLBD). However, bile duct stone removal by ERCP can sometimes be challenging. Difficult bile duct stones (DBDSs) are defined as bile duct stones that cannot be removed using standard ERCP techniques [1]. They include large, multiple, impacted, intrahepatic, and cystic duct stones that cannot be technically grasped using standard devices, such as basket and balloon catheters and mechanical lithotripters. DBDs often require multiple ERCP sessions, which can increase the risk of adverse events (AEs).

Peroral cholangioscopy (POCS) has recently become a useful modality for diagnostic and therapeutic procedures in patients with biliary diseases [2, 3]. Electrohydraulic lithotripsy (EHL) or laser lithotripsy (LL) performed using POCS under ERCP guidance is hence considered to be an option for the treatment of DBDSs.

The new Spy Glass DS Direct Visualization System (Spy DS) (Boston Scientific Corp., Tokyo, Japan), a digital single‐operator cholangioscopy system, was developed with marked improvements in image quality and system setup compared with the first‐generation SpyGlass system. In particular, the relatively high degree of freedom of operation owing to the four‐directional angle and the ability to supply and aspirate water at the same time owing to the independent irrigation ports are very useful for accurate bile duct biopsies and enable less invasive lithotripsy procedures [4].

Recently, the efficacy of POCS‐guided lithotripsy (POCS‐L) for DBDSs has been reported [1]. However, few studies to date have comprehensively evaluated not only its effectiveness but also its safety and the risk factors for AEs. Therefore, in this study, we retrospectively analyzed the effectiveness and safety of POCS‐L for DBDSs in a large patient cohort.

## 2 Methods and Patients

### 2.1 Patients

This study included 132 consecutive patients (82 men and 50 women; median age, 73.0 years [interquartile range (IQR), 68–81 years]) who underwent POCS‐L for DBDSs between December 2014 and July 2023 at Tokyo Medical University Hospital. Patients with surgically altered anatomy (SAA) were excluded from this study. DBDSs were defined as stones that could not be removed using standard stone extraction devices, such as a basket catheter, balloon catheter, or mechanical lithotripter, despite prior papillary intervention with EST, EPBD, or EPLBD. Baseline patient characteristics are summarized in Table 1. The primary indications for therapeutic Spy DS use were common bile duct (CBD) stones (76.5%, *n* = 101) and intrahepatic bile duct (IHBD) stones (23.5%, *n* = 31). Regarding stone number, 76.5% (*n* = 101) of patients had a single stone, 9.8% (*n* = 13) had two stones, and 13.6% (*n* = 18) had three or more stones. The mean maximum stone diameter was 13.8 mm. In all patients, POCS‐L using Spy DS was performed after the resolution of cholangitis. Regarding preprocedural antibiotics administration, the decision was left to the discretion of the attending physician, and there were no clearly defined criteria for administration. Preprocedural and postprocedural data were collected through a detailed review of the patients' electronic medical records. Written informed consent was obtained from all patients. This retrospective study was approved by the Institutional Review Board of Tokyo Medical University (study approval no. T2020‐0349).

**TABLE 1:** Baseline characteristics of patients with difficult bile duct stones.

Variable	Patients (*n* = 132)
Age, years, median (IQR)	73 (68–81)
Male sex, *n* (%)	82 (62.1)
Stone location, *n* (%)	
Common bile duct	101 (76.5)
Intrahepatic bile duct	31 (23.5)
Number of stones, *n* (%)	
1	101 (76.5)
2	13 (9.8)
> 3	18 (13.6)
Stone diameter, mm, median (IQR)	13.8 (5.5–24.5)

Abbreviation: IQR, interquartile range.

### 2.2 Endoscopic Procedures

After visualization of the target stones using Spy DS, lithotripsy was performed using EHL or a holmium: yttrium‐aluminum‐garnet (Ho:YAG) laser (Figure 1). EHL was performed using the Autolith Touch EHL system (Boston Scientific Corp., Marlborough, MA, USA). LL was performed using a Ho:YAG laser system (VersaPulse PowerSuite; Lumenis, Yokneam, Israel). The selection of lithotripsy modality (EHL or Ho:YAG laser lithotripsy) was based on the discretion of the endoscopist according to the anticipated stone characteristics and procedural difficulty. In the authors' institution, EHL was generally used as the first‐line lithotripsy modality. However, in cases in which preprocedural imaging suggested hard stones that were unlikely to be fragmented using EHL, Ho:YAG laser lithotripsy was selected as the initial treatment modality. This study was not designed to directly compare the clinical outcomes between EHL and Ho:YAG laser lithotripsy. All lithotripsy procedures were performed under direct cholangioscopic visualization, and fragmented stones were removed using standard extraction techniques, including basket and balloon catheters. All procedures were performed by endoscopists experienced in pancreatobiliary interventions.

**FIGURE 1:**
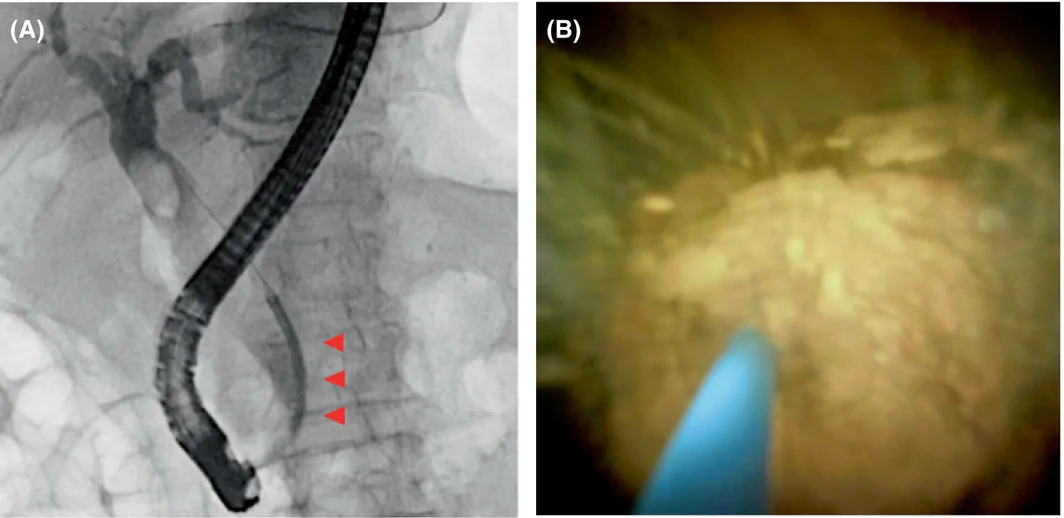
Fluoroscopic and cholangioscopic images of POCS‐L. (A) Fluoroscopic image showing the cholangioscope (red label) being advanced into the bile duct over a guidewire to obtain a frontal view of the stone. (B) Direct cholangioscopic visualization of the bile duct being irrigated with saline, with holmium: Yttrium–aluminum–garnet laser lithotripsy (Ho‐YAG LL) applied to the stone surface to achieve fragmentation (the blue bar indicates the YAG laser).

### 2.3 Outcome Measurements

The primary outcome was clinical success, and secondary outcomes included technical success, procedure time, and procedure‐associated AEs. Clinical and procedural data were collected, including papillary intervention prior to cannulation, postprocedural drainage methods, antibiotic administration, vital signs, laboratory test results, procedure time, stone size and number, and procedure‐associated AEs. Procedure time was determined through a review of video recordings from all endoscopic procedures. Stone size and number were confirmed by cholangiography. For Spy DS‐guided lithotripsy, technical success was defined as successful visualization of the bile duct stones followed by fragmentation using EHL or Ho:YAG laser lithotripsy. Clinical success was defined as complete removal of the bile duct stones. Additional variables analyzed included stone location and diameter, papillary procedures performed prior to Spy DS insertion, use of adjunctive mechanical lithotripsy, and the number of ERCP sessions required to achieve complete stone clearance.

Procedure‐associated AEs were defined and graded according to the American Society for Gastrointestinal Endoscopy lexicon [5]. Postprocedural fever was defined as a body temperature of 38.0°C or higher on the day following the procedure. Cholangitis associated with Spy DS procedures was diagnosed according to the Tokyo Guidelines 2018 [6].

### 2.4 Statistical Analysis

Continuous variables are presented as the mean ± standard deviation or median with IQR, as appropriate, and were compared using the Student *t*‐test or Wilcoxon rank‐sum test. Categorical variables were compared using the chi‐square test or Fisher's exact test. To identify factors associated with adverse events, univariable analyses were initially performed. Variables with a *p* value < 0.10 in the univariable analyses were subsequently entered into a multivariable logistic regression model to identify independent predictors. Statistical analyses were performed using SPSS software (version 26; IBM Corp., Armonk, NY, USA). All tests were two‐sided, and a *p* value < 0.05 was considered to indicate a statistically significant difference between groups.

## 3 Results

Technical success was achieved in 98.5% of the patients (130/132). In 2 patients with IHBD stones, obtaining a frontal view of the target stones was not possible. One patient subsequently underwent endoscopic ultrasound‐guided antegrade stone removal (EUS‐ASR), whereas the other required partial hepatectomy. Among the patients in whom stone removal was attempted using Spy DS‐guided lithotripsy, stone clearance was not achieved in 5 patients (3.8%). This was primarily due to the inability to maintain a frontal view of the stones or because the stones extended into the peripheral bile ducts. Among these 5 patients, 3 underwent hepatic resection because the peripheral IHBD stones were inaccessible endoscopically, and 2 patients required periodic biliary stent replacement due to persistent difficulty with lithotripsy despite multiple EHL sessions. Ultimately, the clinical success rate was 94.7% (125/132) (Figure S1).

### 3.1 Outcomes According to Stone Location

The technical and clinical success rates of CBD stones were 100% and 98%, respectively. For IHBD stones, the corresponding rates were 93.5% and 83.9%, respectively. Technical and clinical success rates were significantly higher in the CBD stone group than in the IHBD stone group (*p* = 0.04 and *p* = 0.01, respectively). The median procedure time tended to be longer in the IHBD stone group than in the CBD stone group (60 [45–88] min vs. 54 [31–70] min, *p* = 0.89). In addition, adverse events occurred more frequently in the IHBD stone group than in the CBD stone group, although the difference was not statistically significant (32.3% vs. 28.7%, *p* = 0.82). Clinical outcomes stratified according to stone location, including procedure time and adverse event rates, are summarized in Table 2.

**TABLE 2:** Clinical outcomes stratified by stone location.

Variable	Overall	CBD stones	IHBD stones	*p*
Technical success	130/132 (98.5%)	101/101 (100%)	29/31 (93.5%)	0.04
Clinical success	125/132 (94.7%)	99/101 (98.0%)	26/31 (83.9%)	0.01
Procedure time, min	56 (31–88)	54 (31–70)	60 (45–88)	0.89
AE	39/132 (29.5%)	29/101 (28.7%)	10/31 (32.3%)	0.82

Abbreviations: AE, adverse events; CBD, common bile duct; IHBD, intrahepatic bile duct.

### 3.2 Procedural Characteristics

EST alone was performed in 50.8% of the patients (*n* = 67), whereas EPBD or EPLBD with EST was performed in 49.2% (*n* = 65). Under Spy DS guidance, EHL was used in 84.8% of the patients (*n* = 112), and Ho:YAG laser lithotripsy was used in 15.2% (*n* = 20). No crossover between EHL and laser lithotripsy was observed. Following lithotripsy, stone extraction was performed using baskets in 68.9% of the patients (*n* = 91), balloons in 55.3% (*n* = 73), and mechanical lithotripters in 53.8% (*n* = 71). Postprocedural drainage consisted of endoscopic nasobiliary drainage (ENBD) in 56.8% (*n* = 75) and endoscopic biliary drainage (EBD) in 22.7% (*n* = 30), whereas drainage was not performed in 20.5% of the patients (*n* = 27). Lithotripsy was completed in a single session in 80.8% of patients (*n* = 106), while complete stone removal within a single session was achieved in 23.8% of all patients (*n* = 31). Lithotripsy required two sessions in 15.2% of patients (*n* = 20) and three or more sessions in 4.5% of patients (*n* = 6). The median procedure time was 56 min (IQR: 31–88 min). Procedural characteristics of POCS‐L are summarized in Table 3.

**TABLE 3:** Procedural characteristics of POCS‐guided lithotripsy.

Variable	Patients (*n* = 132)
Papillary intervention, *n* (%)	
EST	67 (50.8)
EPBD/EPLBD with EST	65 (49.2)
Lithotripsy modality, *n* (%)	
EHL	112 (84.8)
Ho:YAG laser	20 (15.2)
Stone extraction device, *n* (%)^a^	
Basket	91 (68.9)
Balloon	73 (55.3)
Mechanical lithotripter	71 (53.8)
Postprocedural drainage, *n* (%)	
ENBD	75 (56.8)
EBD	30 (22.7)
No drainage	27 (20.5)
Number of lithotripsy sessions, *n* (%)	
1	106 (80.3)
2	20 (15.2)
≥ 3	6 (4.5)
Procedure time, min, median (IQR)	56 (31–88)

Abbreviations: EBD, endoscopic biliary drainage; EHL, electrohydraulic lithotripsy; ENBD, endoscopic nasobiliary drainage; EPLBD, endoscopic papillary large balloon dilation; EST, endoscopic sphincterotomy.

^a^ Multiple devices were used in some patients.

### 3.3 AEs

Overall, procedure‐associated AEs occurred in 29.5% of patients (*n* = 39). Fever occurred in 16.7% (*n* = 22), mild cholangitis in 7.6% (*n* = 10), moderate cholangitis in 3.8% (*n* = 5), pancreatitis in 0.8% (*n* = 1), and gastrointestinal perforation due to EST in 0.8% of the patients (*n* = 1). All patients with postprocedural cholangitis were successfully managed with antibiotics alone. The patient with gastrointestinal perforation was managed conservatively with antibiotics and bowel rest, and no surgical intervention was required. The procedure‐associated AEs are summarized in Table 4.

**TABLE 4:** Procedure‐associated adverse events.

Severity	Adverse event	*n* (%)
All	Any adverse event	39 (29.5)
Mild	Fever	22 (16.7)
	Cholangitis	10 (7.6)
Moderate	Cholangitis	5 (3.8)
	Pancreatitis	1 (0.8)
Severe	Intestinal perforation	1 (0.8)

### 3.4 Risk Factors for Cholangitis After POCS‐L

The risk factors for postprocedural cholangitis were analyzed. The results of the univariable and multivariable analyses are summarized in Tables 5 and 6, respectively. Univariable analyses were performed to evaluate potential risk factors, including age, sex, stone diameter, number of stones, stone location, postprocedural biliary drainage (EBD or ENBD), preprocedural antibiotics administration, and procedure time. A stone diameter of more than 20 mm, absence of postprocedural drainage, and lack of preprocedural antibiotics administration were significantly associated with an increased risk of cholangitis (Table 5). Variables identified as significant in the univariable analyses were subsequently entered into a multivariable logistic regression model. Multivariable analysis demonstrated that a stone diameter of more than 20 mm (odds ratio [OR]: 4.79; 95% confidence interval [CI]: 1.34–17.07; *p* = 0.016) and the absence of postprocedural drainage (OR: 3.73; 95% CI: 1.01–13.7; *p* = 0.048) were independent risk factors for postprocedural cholangitis (Table 6).

**TABLE 5:** Univariable analysis of risk factors for postprocedural cholangitis.

Variable	Total (*n* = 132)	Cholangitis (*n* = 15)	*p*‐value
Age ≥ 75 years	47	5 (10.6%)	0.721
Age < 75 years	85	10 (11.8%)	
Male sex	82	7 (8.5%)	0.23
Female sex	50	8 (16.0%)	
Stone diameter ≥ 20 mm	29	6 (20.7%)	0.021
Stone diameter < 20 mm	103	9 (8.7%)	
≥ 3stones	18	1 (5.6%)	0.365
< 3stones	114	14 (12.3%)	
Intrahepatic bile duct stones	31	3 (9.7%)	0.731
Extrahepatic bile duct stones	101	12 (11.9%)	
No postprocedural drainage	27	6 (22.2%)	< 0.01
Postprocedural drainage	105	9 (8.6%)	
No preprocedural antibiotics	71	10 (14.1%)	< 0.01
Preprocedural antibiotics	61	5 (8.2%)	
Procedure time ≥ 60 min	69	9 (13.0%)	0.429
Procedure time < 60 min	63	6 (9.5%)	

**TABLE 6:** Multivariable analysis of risk factors for postprocedural cholangitis.

Variable	OR	95% CI	*p*
Stone diameter > 20 mm	4.79	1.34–17.07	0.016
No postprocedural drainage	3.73	1.01–13.7	0.048
No preprocedural antibiotics	2.08	0.57–7.69	0.268

Abbreviations: CI, confidence interval; OR, odds ratio.

## 4 Discussion

In this study, we found that POCS‐L using EHL or the Ho:YAG laser has high technical and clinical success rates with an acceptable safety profile for the treatment of DBDSs. To the best of our knowledge, this study included one of the largest cohorts to date evaluating POCS‐L for DBDSs, and is the first to comprehensively investigate risk factors for postprocedural cholangitis.

Previous studies have reported bile duct stone clearance rates ranging from 86% to 100% using POCS‐guided EHL [7, 8, 9]. Recent technological advancements in the Spy DS system, including improved maneuverability, high‐resolution imaging, and efficient irrigation and aspiration capabilities, likely contributed to the improved outcomes observed in this study. Furthermore, the availability of Ho:YAG laser lithotripsy, which enables powerful and precise stone fragmentation, may further enhance treatment efficacy.

In the present study, complete stone clearance was achieved in 94.7% of patients. Cases in which stone removal was unsuccessful were predominantly associated with IHBD stones, highlighting the technical challenges of accessing peripheral bile ducts. This difficulty may be attributed to the anatomical characteristics of IHBD, which are narrower and more tortuous than CBD, thereby limiting cholangioscope maneuverability. In particular, stones located in the posterior branch of the IHBD were often associated with severe ductal angulation, making insertion of the POCS difficult and resulting in technically challenging procedures. Therefore, further subgroup analysis according to IHBD branch may have revealed additional significant differences. However, because of the limited number of patients, IHBD stones were analyzed as a single group. In addition, patients with IHBD stones accompanied by biliary stricture were excluded from this study. Alternative approaches, including EUS‐ASR and partial hepatectomy, were hence required in some patients. In addition, some older patients with particularly hard stones underwent periodic biliary stent replacement instead of definitive stone removal, reflecting the requirement for individualized treatment strategies based on procedural feasibility and patient condition. Recently, advances in endoscopic ultrasound‐guided biliary drainage have expanded the therapeutic options for difficult IHBD stones, and several studies have suggested its utility for DBDSs [10, 11, 12]. Therefore, EUS‐guided interventions may represent a valuable adjunctive or alternative strategy when conventional POCS‐guided lithotripsy is technically challenging.

Cholangitis is the most commonly reported AE associated with POCS, with an incidence ranging from 0% to 14% [13]. In our cohort, the overall AE rate was 29.5%, and cholangitis was the most frequent complication (11.4%). One possible mechanism is the increase in intrabiliary pressure caused by continuous saline irrigation during lithotripsy, which may promote bacterial translocation. Papillary edema following prolonged procedures may also contribute to transient biliary obstruction. Our multivariable analysis identified stone diameters of more than 20 mm and the absence of postprocedural biliary drainage as independent risk factors for cholangitis. These findings suggest that adequate biliary decompression after lithotripsy is essential, particularly in patients with large stones. Indeed, patients who underwent drainage by ENBD or EBD had a significantly lower incidence of cholangitis than those who did not undergo drainage. Regarding drainage strategies, EBD was typically selected when repeated procedures were anticipated, whereas ENBD was preferred when repeat cholangiography was required to evaluate residual stones. In addition, the analysis of postprocedural biliary drainage may have been influenced by selection bias. The decision to place ENBD or EBD was not standardized and was left to the discretion of the attending endoscopist based on procedural difficulty, residual stones, biliary drainage status, and the possibility of additional procedures. Therefore, the association between the absence of postprocedural drainage and cholangitis should be interpreted cautiously because of the potential influence of confounding by indication. Preprocedural antibiotics administration was associated with a reduced incidence of cholangitis in univariable analysis, although this association did not reach statistical significance in the multivariable model. This protective effect may be explained by the prevention or treatment of transient bacteremia resulting from increased intrabiliary pressure during irrigation. Procedure‐related cholangitis occurred in 15 patients, of whom 10 were classified as mild and 5 as moderate according to the American Society for Gastrointestinal Endoscopy lexicon. All cases improved with conservative management without the need for invasive intervention.

Notably, treatment outcomes and AEs were not significantly affected by patient age, suggesting that POCS‐L is both effective and safe even in older patients. However, considering the longer procedure time compared with conventional ERCP, care should be taken to prevent aspiration pneumonia in older patients.

The cases of DBDSs included in this study were those in which conventional ERCP was unsuccessful or was considered unlikely to succeed. Considering the high efficacy and acceptable safety demonstrated in our cohort, POCS‐L should be considered an early treatment option when pretreatment imaging suggests that standard ERCP techniques may be insufficient.

Post‐ERCP pancreatitis is another well‐recognized AE, with a reported cumulative incidence of approximately 3.5% to 10.2% [14, 15, 16]. In contrast, only one case (0.8%) of moderate pancreatitis occurred in our present study, possibly because papillary intervention had already been performed before lithotripsy.

This study has several limitations. First, its retrospective single‐center design introduces potential selection and technical biases. Second, the choice of lithotripsy modality, drainage strategy, and antibiotics use was left to the discretion of the endoscopist, which may have introduced bias. In particular, Ho:YAG laser lithotripsy tended to be used for harder stones identified on preprocedural imaging, which may have introduced treatment‐selection bias. However, the present study was intended to evaluate the overall clinical outcomes of POCS‐guided lithotripsy and was not designed to compare different lithotripsy modalities; therefore, direct comparison between EHL and Ho:YAG laser lithotripsy was not performed. Third, although subgroup analyses according to stone location were performed, the number of patients with IHBD stones was relatively limited, which may have reduced the statistical reliability of separate multivariable analyses for the CBD and IHBD stone groups. Therefore, these subgroup findings should be interpreted with caution. Finally, despite representing a relatively large cohort, prospective multicenter studies are needed in the future to establish standardized treatment strategies and to further validate our findings.

## 5 Conclusion

POCS‐L using the Spy DS system is an effective and safe treatment for DBDS. Postprocedural biliary drainage may help reduce the risk of cholangitis, particularly in patients with stones of more than 20 mm.

## Conflicts of Interest

T.I., T.T., and S.M. have received lecture fees from Boston Scientific Corporation. The other authors declare no conflicts of interest associated with this work.

## Supporting information


**Figure S1:** Flowchart of the therapeutic procedures and outcomes in this study. This flowchart summarizes the therapeutic strategy and outcomes of patients undergoing peroral cholangioscopy‐guided lithotripsy (POCS‐L) for difficult bile duct stones. Technical success was achieved in 98.5% of patients, and complete stone removal (clinical success) was achieved in 94.7%. Alternative treatments, including endoscopic ultrasound‐guided antegrade stone removal or surgical management, were performed in patients in whom endoscopic stone clearance was not achieved. Abbreviations: POCS‐L, peroral cholangioscopy‐guided lithotripsy; EUS‐ASR, endoscopic ultrasound‐guided antegrade stone removal.

## Data Availability

The data that support the findings of this study are available on request from the corresponding author. The data are not publicly available due to privacy or ethical restrictions.
